# Utilizing EEG and fNIRS for the detection of sleep-deprivation-induced fatigue and its inhibition using colored light stimulation

**DOI:** 10.1038/s41598-023-33426-2

**Published:** 2023-04-20

**Authors:** Zeshan Shoaib, Arbab Akbar, Eung Soo Kim, Muhammad Ahmad Kamran, Jun Hyun Kim, Myung Yung Jeong

**Affiliations:** 1 Department of Cogno-Mechatronics Engineering, Pusan National University, Busandaehak-ro 63 beon-gil 2, Geumjeong-gu, Busan, 46241 Korea; 2 Department of Electronic and Robot Engineering, Busan University of Foreign Studies, 65, KeumSaem-Ro 485 beongil, KeumJeong-Gu, Busan, 46234 Korea

**Keywords:** Biomedical engineering, Imaging and sensing

## Abstract

Drowsy driving is a common, but underestimated phenomenon in terms of associated risks as it often results in crashes causing fatalities and serious injuries. It is a challenging task to alert or reduce the driver’s drowsy state using non-invasive techniques. In this study, a drowsiness reduction strategy has been developed and analyzed using exposure to different light colors and recording the corresponding electrical and biological brain activities. 31 subjects were examined by dividing them into 2 classes, a control group, and a healthy group. Fourteen EEG and 42 fNIRS channels were used to gather neurological data from two brain regions (prefrontal and visual cortices). Experiments shining 3 different colored lights have been carried out on them at certain times when there is a high probability to get drowsy. The results of this study show that there is a significant increase in HbO of a sleep-deprived participant when he is exposed to blue light. Similarly, the beta band of EEG also showed an increased response. However, the study found that there is no considerable increase in HbO and beta band power in the case of red and green light exposures. In addition to that, values of other physiological signals acquired such as heart rate, eye blinking, and self-reported Karolinska Sleepiness Scale scores validated the findings predicted by the electrical and biological signals. The statistical significance of the signals achieved has been tested using repeated measures ANOVA and *t*-tests. Correlation scores were also calculated to find the association between the changes in the data signals with the corresponding changes in the alertness level.

## Introduction

The driving process is a demanding, multifaceted, and often perilous task requiring an individual's entire cognitive and physical resources to sustain safe performance. Vehicle driving in drowsy or fatigued brain states distracts the attention and may result in disastrous situations. These situations lead to car crashes and/or fatal accidents involving loss of multiple lives and/or causing injuries and in some cases life time disabilities^1^. National Highway Traffic Safety Administration (NHTSA) USA has compiled car accident data during fatigued or drowsy states which shows alarming figures of 2.4% of total car crashes due to drowsy state driving through years 2011 to 2015. These statistics reveal a huge number of human lives lost due to drowsy driving almost comparable to the drunk driving fatalities^2^. Extended working hours^3^, sleep deprivation^4^, and medical disorders^5^ are a few of the many possible contributors to this compromised alertness state. Generally drowsy brain state is defined as loss of processing efficiency of human brain. There are two possible methodologies to avoid fatal accidents while driving under drowsy or fatigued brain state. The first strategy is preventive approach in which the driver of a vehicle is continuously monitored through different types of sensors. These sensors generate several alerts as soon as the driver falls to the drowsy brain state. While in the second technique, the vehicle is controlled through an automatic software based safety system which safely parks the car on road side in case of drowsy or fatigued state. The preventive measure technique is beneficial and has attracted several researchers to propose algorithms and schemes to alert the driver or help in reducing his drowsy state.

As drivers’ fatigue has a direct relation with an increased risk of road accidents, therefore, automobile industry has invested in the development of preventive equipment to reduce car crashes^6^. The automobile industry/companies have invested huge amount of money and technical resources to develop automatic intelligent systems that timely alert drowsy drivers and take other automated steps^7^. A leading automobile company, Toyota Motors has developed, ‘‘Toyota Safety Sense’’^8^, a distinguishing system that responds to cases of driver heedlessness. The system works with two main features;

- A pre-collision detection system that safeguards the vehicle from collisions by employing visual and audio alarms, and, a system that automatically applies brakes to avoid crashes after detecting the driver in a drowsy state.
- The system is powered by a lane assist mechanism that generates audio / visual indications in cases of lane deviations without the use of vehicle indicators. In case of unresponsiveness to these indications, the system automatically adjusts the position of the steering wheel.

Hyundai motor company introduced the ‘‘Driver Attention Warning’’ system, which takes the steering wheel’s position, the angle of torque applied to it, lane position, and the driving time to determine the driving pattern. The system displays a coffee mug and sounds an alarm if it detects the driver in a drowsy state^9^. ‘‘Attention Assist System’’ developed by the Mercedes automobile company observes sensors installed in the steering wheel, acceleration and braking patterns of the car, road condition, etc. to determine the driver's mental state^10^. During a particular trip, the system generates a driver’s profile with certain thresholds based on the above-mentioned factors. In case of a considerable deviation from the generated profile, the system renders a warning sign and beeps. Ford Motors have also introduced a ‘‘Driver Alert System’’ that utilizes a pinhole camera to record the moving patterns of the car. Whenever the driver is deviating from a specific lane, a vibration in the steering wheel is generated to alert the driver and eventually lane is automatically adjusted if the driver is found to be drowsy^11^. A ‘‘Driver’s Drowsiness detection system’’ is equipped in automobiles from Bosch company that analyzes steering movement, the car’s steering angle, and the velocity profile to alert the driver in a drowsy brain state^12^. All these famous automobile companies have installed drowsiness indicator systems but there is no cognitive mechanism developed in the industry yet.

A more direct and objective assessment of alertness level is provided by evaluating physiological parameters, those can be assessed continually. Fatigue-related signals may also be monitored using functional Near-Infrared Spectroscopy (fNIRS), Electroencephalogram (EEG), and functional Magnetic Resonance Imaging (fMRI)^13–16^. Several studies have employed fNIRS for drowsiness detection and also to discover association between neuro-physiological responses and driver’s mental state. Cai et al. utilized fNIRS to evaluate fatigue level by scanning the parietal-occipital brain region^17^. Data from multiple brain regions using fNIRS was acquired by Chuang et al. to discriminate the alert and drowsy brain states based on dHBO power^18^. Karageorghis et al. measured fNIRS’s hemodynamic signal showing that music may help to avert fatigue^19^. Using EEG, Barua et al. analyzed sleepiness while driving complemented by EOG^20^. Wang et al. in a study utilized EEG in real driving task to evaluate the frequency bands’ PSD and sample entropy for the detection of drivers’ fatigue^21^. In a simulated driving task involving 15 male subjects, EEG signals were obtained by Chen et al., for drowsiness detection^22^. Yang et al. utilized behavioral and EEG data from 52 participants in a simulated driving experiment to classify drowsy and alert brain states^23^. Several studies have employed fMRI for the task because of better spatial resolution than EEG or fNIRS^24^. Using fMRI, Allen et al. examined the brain network connectivity patterns in open-eyes and closed-eyes states to highlight brain’s sub-regions of interest for drowsy and alert states^25^. In a study based on fMRI, with divided-attention task, Drummond et al. detected an improved activation in the pre-frontal region in the drowsy state^26^. The fNIRS and EEG modalities have attracted many research groups because of easy setup, portability, and cost effectiveness^27,28^ as compared to fMRI because its experimentation is difficult, requires certain pre-requisites, and is time taking^29^. Apart from EEG, fNIRS, and fMRI there are several other physiological parameters that can help in detecting the drowsiness level of human brain. Such factors include Epworth sleepiness scale, a questionnaire based method of estimating drowsiness/sleepiness levels^30^. Karolinska Sleepiness Scale (KSS) test is another sleepiness quantification self-reporting test^31^. Another important physiological parameter is electrocardiogram (ECG), it can continuously measures heart rate (HR) and heart rate variability (HRV), where a decrease in average HR of a subject predicts drowsiness^32^. Electro-Oculogram (EOG) monitors the eye movement of a subject and estimates alertness level^33^. Some researchers have presented different head movement characteristics for normal and drowsy persons, therefore it may be used to classify sleepy/drowsy states^34^. Eye blinking patterns and the gap between eyelids are also helpful in distinguishing between an awake and a drowsy person^35–37^.

Exposure to light has shown certain effects on various human behaviors and diseases. Light therapy is a famous and effective technique to treat several human psychological, medical, and behavioral conditions^38,39^. Exposing bright light has been found to treat seasonal affective disorder^40^. Several other types of depression have also been treated with bright light such as bipolar depression, chronic depressive disorder, ante-and postpartum depression, and behavioral disturbance etc.^41^. A study has shown that blue light exposure is effective for treatment of inflammatory acne^42,43^. Light emitting diode based low level light therapy (LED-LLLT) has proved an enhanced blood flow in epidermis and dermis skin layers which attracted plastic surgeons to treat the aging face problem using this technique^44^. Lack et al. showed that exposure to bright morning light for 1 week improves the sleep quality and daytime feelings among people with sleep onset insomnia with a delayed circadian rhythm^45^. Moreover, human brain also shows changes in signals with exposure to different light conditions. Light exposure directly affects how well a person can think and process information because of its effects on nonvisual functions in the brain^46^. Blue light influences cognitive processes that are regulated by a melanopsin-based photoreceptor system controlling circadian cycles^47^. Different ambient lighting conditions may result in reduction of stress and improving driving performance in particular environments like long tunnels^48^. Several other studies also analyzed specific light intensities exposure and colors to humans lead to peculiar outcomes such as improved response times, change in precision metrics, and increased alertness^49,50^. Additionally, some other neuroimaging studies concluded that subcortical structures of the brain, such as the brain stem, hypothalamus, and thalamus, which are connected to wake promotion, are affected by light exposure^51,52^.

This study proposes a drowsiness reduction scheme by analyzing the changes in neurological and physiological signals of participants with the exposure of different colors of light. EEG and fNIRS brain imaging modalities were utilized to acquire neurological data of participants from the prefrontal and visual cortices brain. 31 healthy participants were divided in a control and a healthy group. All the participants were involved in several experiments asking them to drive in a simulated driving environment for 50/60 min in different colored lights exposures. The experimentation time was cleverly selected to increase the probability of falling drowsy. The study revealed that in case of blue light exposure to a sleep deprived participant, there is a significant increase in HbO, and the beta band power response. On the contrary, the results indicated that in case of red and green light exposures, there was no considerable increase in HbO and beta band power. Additionally, other physiological signals acquired such as heart rate, eye blinking, and self-reported KSS scores also indicated the same findings as projected by the electrical and biological signals. Repeated measures ANOVA test and t-tests were utilized for determining the statistical significance of the signals attained. To highlight the association of the observed changes in the data signals (HbO and beta band) with the changes in the alertness level (KSS scores), corresponding correlation values were also reported.

## Material and methods

**Figure 1:**
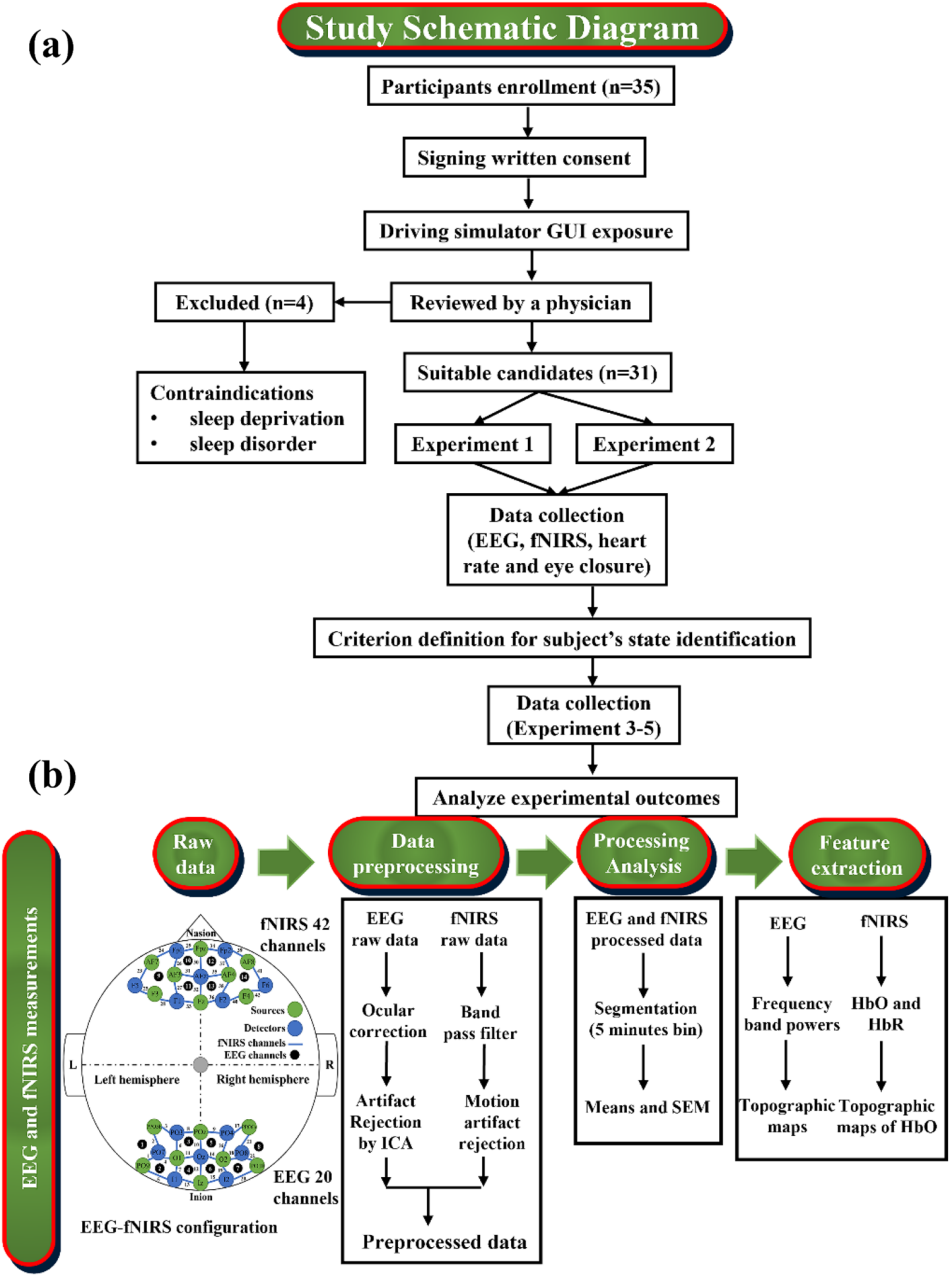
Study schematic for the reduction in fatigue level: (**a**) Flow chart of the experimentation, data collection and analysis procedure of the study. (**b**) Electrical and optical collection of data from subjects’ brains and subsequent data preprocessing and relative feature extraction to generate topographic images resulting in awake/fatigued states of the brain. The details of the schematic and feature extraction process are provided in the Materials and Methods section.

The effect of different colors of light (red, green and blue) illumination causing an increase/decrease in alertness level was studied. This is an investigative study to evaluate the neuronal synchronization, electrical activity, cerebrovascular function and functional connection of sleep-deprived subjects exposed to different colors of light. The primary hypothesis in this work is that different colors of light may effect biological and electrical signals enabling to indirectly measure the drowsy/alert cognition level. Therefore, this study employed advanced analytical and signal processing approaches to extract useful information regarding fatigued/awake states of the human brain caused by different colors of light. The schematic of the study and EEG and fNIRS data processing analysis workflows are depicted in Fig. 1a, b, respectively. The key variables selected for analyses in this study are shown in Table 1.

**Table 1:** Key variables and their units used in this study.

Variable	Unit
Colors of light (red, green and blue)	Wavelength (nm)
HbO	Concentration changes (mM)
HbR	Concentration changes (mM)
Frequency band powers (alpha, beta, theta, delta, gamma)	μV^2^
Eye closure	Unit/sec
Heart rate	Beats per minute (B/M)

### Participants

In this study, 31 drivers were selected for the different colored light exposure experiments in a simulated driving environment. The goal of the study and the experimental protocol were described to the participants as they arrived for the experiment. An informed consent was obtained from all participants in advance. Soon after the subjects signed the consent form, they were advised to practice driving tasks to become accustomed to the system. Participants were prohibited from drinking alcohol, coffee, or tea one day before the experiment. A physician evaluated the subjects and people who could not be deprived of sleep due to certain medical conditions (e.g., epilepsy, diabetes, psychiatric disorders), those who exhibited sleep disorder symptoms or had a medical problem, or those who were on drugs that would adversely affect mental performance (e.g., neurological disease, psychotropic medication) were excluded from the study. Additionally, participants who needed vision glasses were excluded from the study. To assure participants' suitability, the following responses were also recorded:

- Driving simulator sickness susceptibility (as measured by the Motion Sickness Susceptibility Questionnaire),
- Last night's sleep quality (on a scale of 1 to 7),
- Routine coffee intake (never, seldom, one or two cups per day, more than two cups per day),
- Driving (highway) frequency (sometimes, several times a month).

To improve the study's performance, participants' responses, such as personal information and routine data, were saved and analyzed for the purpose of the study. The demographic data of the subjects are shown in Table 2. The study was designed and performed according to the latest version of the Declaration of Helsinki, and was approved by the regional Research Ethics Committee (Pusan National University, Busan).

**Table 2:** Demographic variables of the subjects in this study.

Variables	Categories	Count	Mean ± SD	Percentage
Participants	Male	24		77.4
	Female	7		22.6
Age (years)	18–20	4	19 ± 1	12.9
	20–25	13	22.3 ± 1.5	41.9
	25–30	11	26.9 ± 2.2	35.5
	30–35	3	33.4 ± 1.1	9.7
Education (years)	≤ 14	5	13.1 ± 0.5	16.1
	14–16	10	15.3 ± 0.3	32.3
	16–20	16	17.8 ± 2.4	51.6
Sleep hours (per day)	6 or less	14	5.2 ± 0.4	45.2
	7–8	11	7.1 ± 0.2	35.5
	8–9	6	8.09 ± 0.4	19.3
Exercise	Sometimes	12		38.7
	Often	19		61.3
BMI (kg/m^2^)	≤ 20	8	18.6 ± 1	25.8
	20–24	21	22.3 ± 1.4	67.7
	Overweight	2	25.7	6.5
Driving experience (years)	≤ 5	13	3.6 ± 1.1	41.9
	5–10	18	6.8 ± 1.8	58.1
Penalties (traffic offences in last 2 years)	Never	19		61.3
	1–3 times	12		38.7

*The variables estimated through questionnaire.

### Instrumentation

#### EEG recording

Throughout the experiment, continuous EEG data were collected concurrently with fNIRS using a 32-channel ActiCap equipped with a BrainAmp DC amplifier (Brain Products GmbH, Gilching, Germany). The following electrodes were utilized to record EEG data: PPO9 h, POO9 h, POO1, OI1 h, POO2, OI2 h, POO10 h, and PPO10 h in the visual cortex and AFF5 h, AFp1, AFF1 h, AFp2, AFF2 h, and AFF6 h in the prefrontal cortex. The electrodes were positioned between the fNIRS sources and detectors in such a way that the EEG electrodes were positioned as closely as possible to the usual 10–20 arrangement. All electrodes were grounded to a passive electrode and referenced to a common mode sense electrode. The skin under the reference electrodes was cleaned and abraded, and the Ag/AgCl electrodes were prevented from having an impedance over 5kOhm (Impedance < 5kOhm). The sampling rate for EEG was set at 500 Hz. Two ECG channels (left and right chest) were used to obtain the HRs of each subject during the course of the experiment. The participant was monitored via a camera, which was placed at a comfortable distance to observe both the subject's activity and their facial expressions. When a participant closed his or her eyes for more than five seconds, it was counted as an eye closure (EC), and complete video was used to calculate the overall EC count.

#### fNIRS recording

Participants' brain functional data were measured using a NIRScout fNIRS system (NIRX Medical Technologies, LLC, Germany). During the experiment, individuals' HbO and HbR concentrations were continuously recorded, which corresponded to the brain functional data. The fNIRS system employs two different wavelengths of infrared light (785 nm and 850 nm) monitored at a frequency of 3.81 Hz. The visual cortex and prefrontal cortex were designated as regions of interest. For experimentation, several optode-probe sets were mounted to a head cap to cover the two cortices.

One of the optode-probe sets (consisting of eight sources and seven detectors, yielding 22 channels) was intended to cover the participant's visual cortex. The other optode-probe set (which included eight sources and seven detectors and generated 20 channels) was designed to cover the prefrontal cortex. According to the international 10–20 system, the probe sets were meticulously registered. Proper measurements and refinements ensured precise optode placement among all participants. To obtain a suitable signal-to-noise ratio, proper contact between optodes and skin was guaranteed.

#### Exposure to colored light

The red, green, and blue light colors were emitted using light-emitting diode (LED) light sources. A total of 9 LED lights were installed in the experimental room, which could be controlled through a control system in addition to the experimentation room. Three of these LED lights were red (A19 3 W-R, ILSHIN Vitron, Gyeonggi-do, KOEA), 3 were green (A19 3 W-G, ILSHIN Vitron, Gyeonggi-do, and KOEA), and the remaining 3 were blue (A19 3 W-B, ILSHIN Vitron, Gyeonggi-do (A19 3 W-B, ILSHIN Vitron, Gyeonggi-do and KOREA). A CCD camera (DCU224C, Thorlabs, Inc., USA) was utilized to capture the broad spectrum of spectral peaks produced by the light control system, which generates three distinct colors of light. Each light wavelength was measured using the CCD camera and the chromaticity diagram. To determine the wavelengths of light present in the room, the CIE 1931 colorimetric chromaticity diagram was used. The spectral peaks for the three primary colors of light were as follows: red light with a wavelength of 625 nm (full width at half maximum (FWHM) of 17 nm), green light with a wavelength of 520 nm (FWHM of 30 nm), and blue light with a wavelength of 455 nm (FWHM of 22 nm).

#### Driving simulation

A driving environment was simulated using a computer-based driving simulator. The driving simulator system comprised a steering wheel equipped with a brake, an accelerator and a comfortable chair. There was a large display screen at a distance of 1.5 m from the person sitting in the chair. Special software designed for commercial driving simulations was used during the experiments (Gran Turismo 5 on a PlayStation 3). Using curved and straight roads, tiresome 50- and 60-min drive experiments were performed. A rural two-lane road with a lane width of 3.75 m, a right lane boundary, and a smooth randomized curvature was used as a driving scenario. Furthermore, participants were instructed to maintain a speed between 60 and 80 km per hour (kph). A vibratory mechanism was set in the steering wheel to alert the drivers and to prevent them from falling completely asleep whenever the car impacted a crash barrier. After every 16 km of track, the road pattern was repeated. Only 1 experiment in one night was conducted and every participant was provided with a 7-day wash out period before his next experiment. For example, if Experiment 2 on Participant X was performed in the night of Day-1, then it was followed by a 7-Day rest or washout period, and the next experiment (e.g. Experiment 3) on Participant X was carried out in the night of Day-8 on the same time as of his Experiment 2.

### Experimental protocol

Five distinct experimental groups were designed to collect data from the participants. The purpose of such a diverse experimental design was to acquire ample raw data from cortical signals in the fatigued and alert states, as well as to assess the effects of colored light on these brain states. The first two experimental protocols (Experiment 1 and Experiment 2) were developed to extract criteria values for alert and fatigued brain states.

#### Experiment 1 (Without sleep deprivation (considered an awake state))

In experiment 1, the participants were instructed to drive on a simulator for 50-min in awake state, in a sound-proof room, controlled temperature at 26°Celsius, and dim light conditions. Sitting with dim lights and, participants in this experiment were instructed to drive on a simulator for 50 min. It was ensured that the subjects had a good night sleep of 7.9 h ± 0.5 (mean ± SD). Each day, in the morning, only one such experiment was conducted. The likelihood of falling asleep between the times of 02:00 and 06:00 and between the times of 14:00 and 16:00 is three times greater than it is at 10:00 or 19:00, respectively^53,54^. The experimental periods were chosen to best match the experimental needs, incorporating the times with the lowest risk of sleepiness (in the morning) for the awake-state experiments and times with the highest risk of sleepiness (middle of the night) for the sleep deprived-state experiments. During the driving task, after every 10-min session, a self-reported level of alertness was recorded using the KSS score. Participants scheduled for the experiment on a particular day were advised to report to the lab the evening before the trial day. To ensure good quality sleep, they were provided with a pleasant sleeping area inside the laboratory. Furthermore, participants were strictly advised to abstain from alcohol and other carbonated/caffeinated beverages for 24 h prior to the experiment.

#### Experiment 2 (With sleep deprivation)

After completing the awake-state criterion experiments successfully, participants were ready for the fatigue-state criterion experimentation to be set up in the same lab. For these experiments, it was ensured that each participant underwent continuous sleep deprivation for 22 ± 0.5 h (mean ± SD. Participants carried out the driving task for 50 min using the driving simulator keeping the environmental settings the same as in Experiment 1, i.e., soundproof and air-conditioned room with temperature set to 26°Celsius in dim light. As before, participants reported to the lab one day prior to the experiment and were kept awake by engaging in different activities, such as reading, watching movies, and playing games, under the supervision of a research assistant. During the experiment, the KSS score was used to assess and record subjective alertness level every ten minutes. One such experiment was conducted in dim light each day exactly as the awake state experiments. To provoke maximized sleepiness symptoms, these experiments were conducted at midnight.

#### Experiments 3–5 (Colored light exposure under sleep-deprivation conditions)

The major study experiments were designed for participants in a sleep deprived state and then by shining the luminance of colors (red, green, and blue) in their environment. Participants were asked to perform a driving activity using the driving simulator for 60 min under sleep-deprivation conditions, which has already been explained. The total experiment time in each of Experiments 3–5 was divided into 3 subblocks. The main block was preceded and succeeded by 5-min subblocks with dim light conditions. However, the 50-min main block was the period where the surrounding light color was changed. In short, 5 min of test data were captured under dim light both before and after the light luminance block, whereas 50 min of data were collected within the light luminance settings. In Experiments 3, 4, and 5, participants were exposed to red, green, and blue light, respectively. The KSS score was recorded during the main block with the colored light exposure time every 10 min while participants were performing the driving task.

### Preprocessing and other measures

#### EEG preprocessing

The data acquired through the EEG system were analyzed and processed using EEGLAB and MATLAB 2012a (The MathWorks Inc., Natick, MA, USA). A sixth-order Butterworth bandpass filter between 1 and 50 Hz was used to filter the data. The EEG signal was then decomposed into 14 independent components using independent component analysis (ICA). By checking each ICA component manually, components with high percentage of artifacts such as eye blinking and motion artifacts were screened for elimination. Extra care was taken in eliminating only those components having a frequency less than 8 Hz and peaks. On average 2–3 components were eliminated in most of the cases with a maximum of 5 components in a few participants. The remaining components with brain signal greater than 90% were utilized to generate a processed EEG signal. The EEG sample rate gave enough time resolution to capture the frequencies we were expecting to find in the scalp EEG. Finally, the processed EEG data were decomposed into five frequency bands—the delta (1–3 Hz), theta (4–7 Hz), alpha (8–12 Hz), beta (13–29 Hz) and gamma (30–50 Hz) bands. The power spectral density of each band was computed. The power spectrum was computed using the MATLAB function pwelch with a hamming window size of 500 (data points) and a half-length window size of 250 for the overlap (data points). The wavelet transform (Morlet wavelet) with 0.5 frequency step was used to analyze the EEG-band powers. Outliers were removed using two sigmas, and the mean power of all data was calculated.

#### fNIRS preprocessing

We processed the optical data using HOMER2 software^55^. The software utilizes a GLM-based methodology to extract useful information related to the experiment. To annihilate heart-rate artifacts (~ 1 Hz) but to conserve human respiration frequency component (0.2–0.4 Hz) or Mayer waves (0.1 Hz) during the hemodynamic measurement, a low-pass filter was designed for fNIRS at a 0.5 Hz cutoff frequency. Using the modified Beer–Lambert Law (MBLL)^56^, the concentration changes of HbO and deoxyhemoglobin (HbR) were calculated from the signals. Between a source and a detector, the MBLL formulates an exponential attenuation of light along a channel whose effective length is a multiple of the source-detector separation. The differential path length factor (DPF) is dependent on the wavelength of the light emitted by the source and is multiplied by the source-detector separation to obtain the effective path length. It is assumed that HbO and HbR were the only light absorbers in the setup. For the 785-nm and 850-nm wavelengths, extinction coefficients (units cm^-1^ M^−1^) of 1486.6 and 2526.4 were used for HbO, whereas 3843.1 and 1798.6 were used for HbR. The corresponding values of DPF for HbO and HbR were 7.25 and 6.38, respectively.

#### KSS score and other physiological parameters

A modified version of the KSS score was used to obtain subjective evaluations of sleepiness. This KSS version requires the participant to speak out a number between 1 and 7, and each number corresponds to the real-time alertness level (1 = very alert, 2 = alert, 3 = neither alert nor sleepy, 4 = some signs of sleepiness, 5 = sleepy, but no effort to remain awake, 6 = sleepy, some effort to keep alert, 7 = very sleepy, great effort to keep alert, fighting sleep). Participants were asked to review the KSS scale while driving during every driving session. Prior to any experiment, it was ensured that the participant understood what the KSS scale was, and any doubts were clarified. It was also made clear to the participants that they would be asked about the KSS scale value corresponding to their alertness level at the commencement of the experiment and at intervals of 10 min. They were requested to report a number estimating their alertness level at that time. It is quite logical that these sleepiness ratings came from the subjects themselves because it is a subjective measurement that is inaccessible to others.

The ECG data were bandpass filtered between 0.1 and 30 Hz and were de-trended to remove baseline deviations. To measure a subject's HR or to predict any abnormality in heart functionality, the QRS-wave is often utilized. Specifically, an R-peak simply retrieved with an adjustment of the ECG magnitude threshold clearly identifies the HR of the subject. The frequency of these R-peaks was determined for one minute to evaluate HR per minute.

### Statistical analysis

A point-by-point comparison was performed among the time samples of the colored light experiment with the corresponding time signals of the first two experiments. Data from all bio-signals were segregated into 5-min bins except for the KSS scores, which were evaluated in 10-min bins. The mean and standard deviation (SD) for every segment were calculated to visualize and analyze the changes in parameters at the group level. The measured data were verified for normality using the Kolmogorov–Smirnov normality test. Repeated measures ANOVA (RM-ANOVA) was used to compare parameters observed between the different experimentation conditions. The RM-ANOVA test indicates that whether any groups have significant differences without listing the individual groups with the difference. So, further analysis is required to know which group is significantly different from the other. A post hoc analysis using Bonferroni confidence interval adjustments was performed to analyze the groups that are significantly different among each other. The above explained test sequence was successively repeated for data acquired from both brain interfacing modalities i.e., fNIRS and EEG and the physiological parameters i.e., HR, EC, KSS. These test results aided the authors to analyze the differences between participant’s brain states for exposing each color of light. The findings of the study are depicted in figures with a significance level of 5% for all analyses.

### Correlation analysis

We performed a correlation analysis between the fNIRS and EEG signals with the KSS scores of the participants. Here KSS scores are used as the quantitative level of alertness as reported in a number of published studies, and correlate these scores with fNIRS and EEG signals acquired after different color light stimulations. We selected distinct slices of the fNIRS and EEG signals for color light exposure experiments with different levels of neural activation and calculated their correlation with KSS scores for the respected experiments. Correlation coefficient between these signals are calculated using Pearson’s correlation coefficient (r) that may be described as:

1 $$r_{XY} = \frac{{\mathop \sum \nolimits_{i = 1}^{N} \left( {x_{i} - \overline{x} } \right)(y_{i} - \overline{y})}}{{\sqrt {[\mathop \sum \nolimits_{i = 1}^{N} \left( {x_{i} - \overline{x}} \right)^{2} \mathop \sum \nolimits_{i = 1}^{N} \left( {y_{i} - \overline{y}} \right)^{2} ]} }}$$

where $\overline{x }$ denotes the mean value of the first signal ${x}_{i}$ and $\overline{y }$ is the mean of the second signal ${y}_{i}$. The value of ${r}_{XY}$ varies between − 1 and 1. A perfect negative correlation is represented by a value of − 1 and a perfect positive correlation is represented by a value of 1. Whereas ${r}_{XY}=0$ represents that both the signals are completely uncorrelated.

## Results

### Identification of the subjects’ state

**Figure 2:**
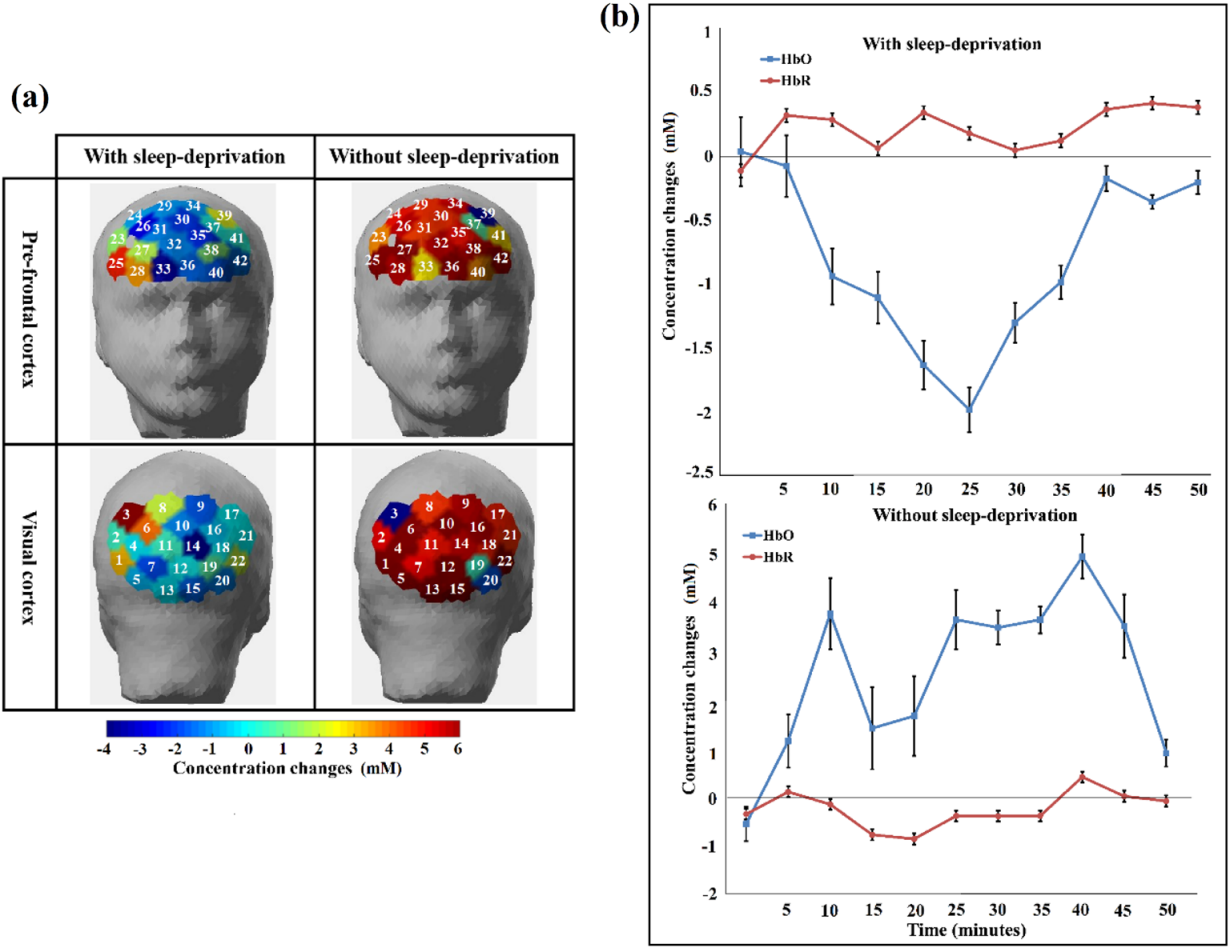
(**a**) The four distinct template maps that best explain channel-wise changes in group mean HbO potential topographies across sleep-deprived (left two in figure) and non-sleep-deprived (right two) conditions in the prefrontal (upper two in figure) and visual (lower two) cortices. Blue represents a decrease in HbO response, while red illustrates an increase in HbO response. (**b**) Average (group-level of all participants) concentration changes in cerebral oxygenation (HbO and HbR) (mean ± SD) during a 50-min drive in a simulator under sleep-deprived conditions (upper in figure) and non-sleep deprivation (lower).

MBLL was used to estimate the time course of changes in the relative concentrations of HbO and HbR. Figure 2a depicts the channel-wise activation in targeted brain regions (topographic maps), showing only the HbO concentration changes. Figure 2b depicts the mean ± SD values of HbO and HbR concentration changes in the 31 subjects for 42 channels in both the sleep deprived (upper in figure) and awake (lower in figure) states.

We observed a significant difference (*p* < α_critical_) in the activation evoked by both conditions in either brain region for HbO. A driver in an alert state utilizes all the required brain functionalities, which results in high oxygen consumption and higher cerebral blood flow. This high oxygen consumption by the brain is represented by an increase in HbO. However, due to diminished brain involvement during the fatigued state, lower levels of HbO were observed.

**Figure 3:**
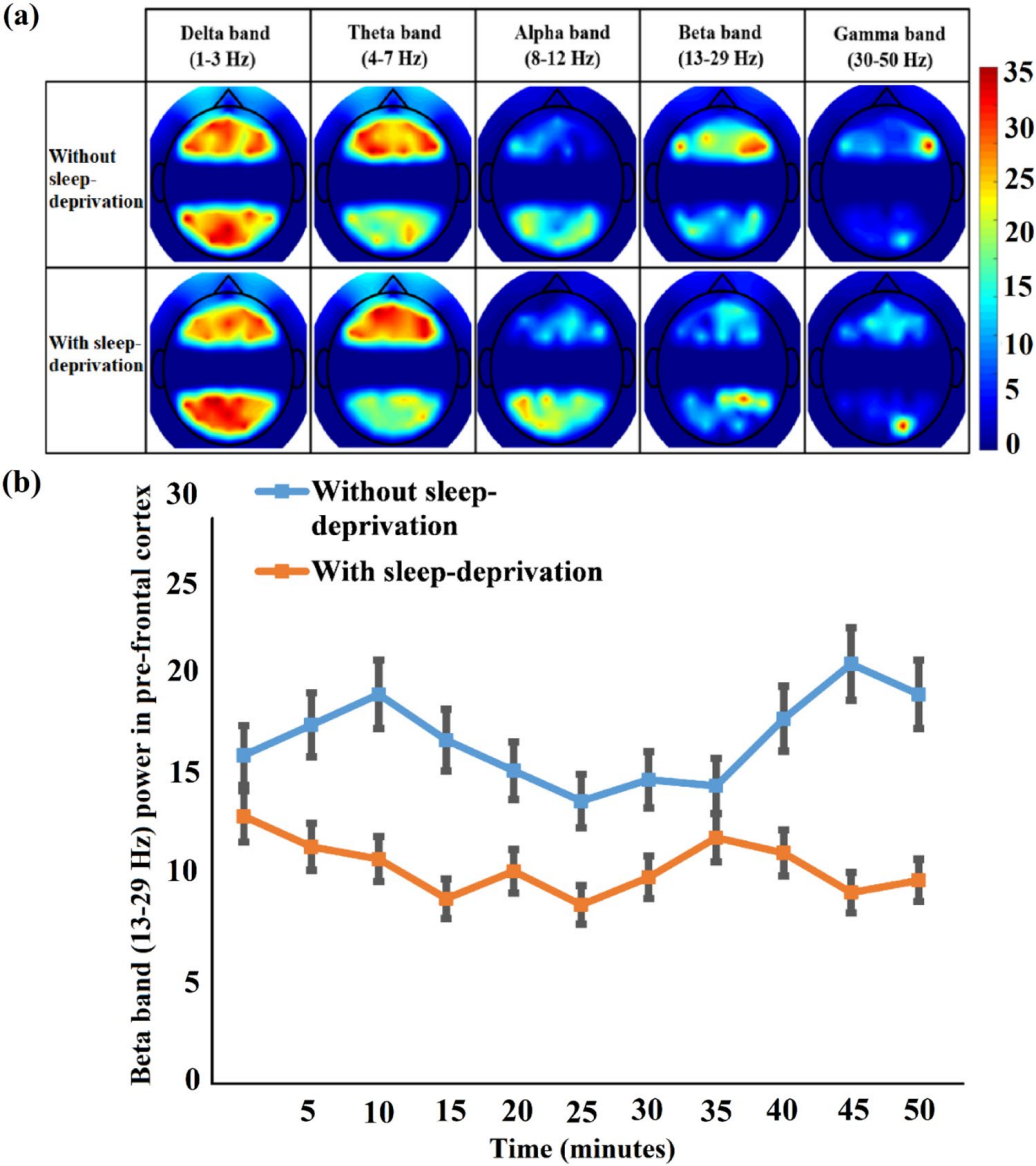
(**a**) The average topography of spatial distributions of the EEG band powers that best explained the changes in band powers across the sleep-deprived and non-sleep-deprived conditions. (**b**) Average normalized time course values of beta EEG band (mean ± SD) under sleep-deprived and non-sleep-deprived conditions for pre-frontal cortex. Drastic discrepancies appear in the EEG beta band across these two conditions.

Figure 3a presents the averaged topographies of all 31 subjects' relative EEG frequency band powers over 14 channels for each condition. Figure 3b represents the mean ± SD values for relative EEG beta band power for the pre-frontal cortex region, which is measured after every 5-min interval during a 50-min driving activity for both the sleep-deprived and non-sleep-deprived conditions. Normalized values were applied to accommodate the variability in the diverse amplitude of beta spectral band power. Significantly different effects of each condition (*p* < α_critical_) were obtained for EEG band power in the frequency range 13–29 Hz (beta band power). However, other frequency band powers had similar effects (*p* > α_critical_) under both sleep-deprived and non-sleep-deprived conditions. A decrease in beta band power over the prefrontal region was observed in the sleep-deprived condition compared to that of non-sleep-deprived subjects, i.e., the beta band power in the non-sleep-deprived condition was activated to a greater degree in the prefrontal region than it was in the sleep-deprived condition. We also investigated other frequency band power values—delta, theta, alpha, and gamma—topographically and found that none of the topographies differed statistically between driving conditions.

**Figure 4:**
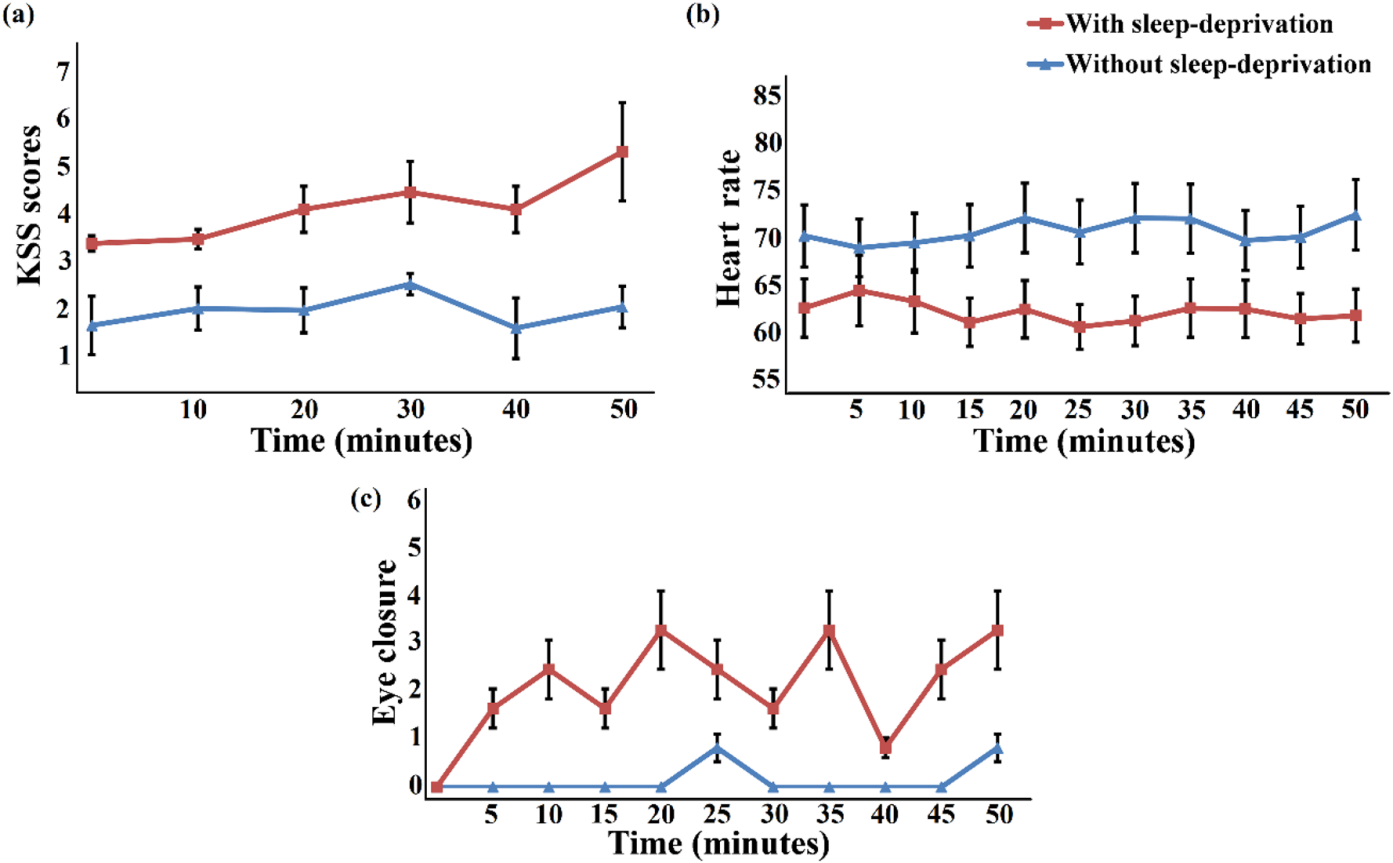
KSS scores, HR, and EC. (**a**) KSS scores (mean ± SD) of 5 time periods (10 min of each period) (x-axis) during a sleep-deprived and non-sleep-deprived 50-min drive in a simulator. (**b**) Average HRs (mean ± SD) of 5-min bins (x-axis) across all the participants under sleep-deprived and non-sleep-deprived conditions during a 50-min drive in a simulator. (**c**) Average EC rates (mean ± SD) of 5-min bins (x-axis) across all participants under sleep-deprived and non-sleep-deprived conditions during a 50-min drive in a simulator.

During both criterion experiments (with sleep deprivation and without sleep deprivation), a self-evaluation test of alertness level was also performed every 10 min using the KSS score, as shown in Fig. 4a. The results show considerably higher fatigued levels for the sleep-deprived subjects during the experiment as compared with those of the non-sleep-deprived subjects. The significant differences between the two conditions confirm that sleep-deprived and non-sleep-deprived subjects reacted differently in their perception of alertness level during the drive. Figure 4b shows the average HR acquired from the ECG signal peaks over the entire 50-min driving period under both conditions. Participants in the sleep-deprived condition exhibited significantly lower HRs than those in the non-sleep-deprived condition. Additionally, a relatively higher rate of EC was observed in the sleep-deprived subjects, and the differences were statistically significant, as shown in Fig. 4c. In the authors’ opinion, this high EC rate can be associated with the driver's struggle to overcome fatigue and stay awake while driving, as evident from the video recordings analyses. Statistical significance among the parameters (KSS, HR, and Eye Closure) was tested for the 2 underlying conditions i.e. with sleep deprivation condition and without sleep deprivation condition using t-test. The resulting p-values obtained for KSS, HR, and Eye closure show significant differences of these parameters in both conditions with *p*-value < α_critical_.

In summary, driver fatigued state has been found to be characterized by decreased beta band power and HbO, increased HbR and KSS scores and decreased HR with more than five seconds of EC. To determine the subject's current state, we employed EC, HR, EEG frequency band powers, and blood oxygenation levels as a feature set. The ECG peaks were counted to determine HR. To determine the number of ECs, subjects were monitored with a video camera to count the number of times they closed their eyes for more than five seconds. The EEG frequency band powers were averaged through 14 EEG channels, and the oxygenation level was averaged through 42 fNIRS channels. The abovementioned five parameters were computed for each minute during 50 (1st and 2nd types of experiments) and 60 (3rd to 5th types of experiments) minutes of measurements. An awake state was determined if a subject had a lower EC rate and KSS score and an increased HR, beta band power and HbO level. In contrast, a sleep deprived state exhibited constant or opposite parameter values. The study chose 5-min EEG and fNIRS recordings in both the awake and sleep deprived states for detailed analysis.

### Effects of different colors of light on alertness level

**Figure 5:**
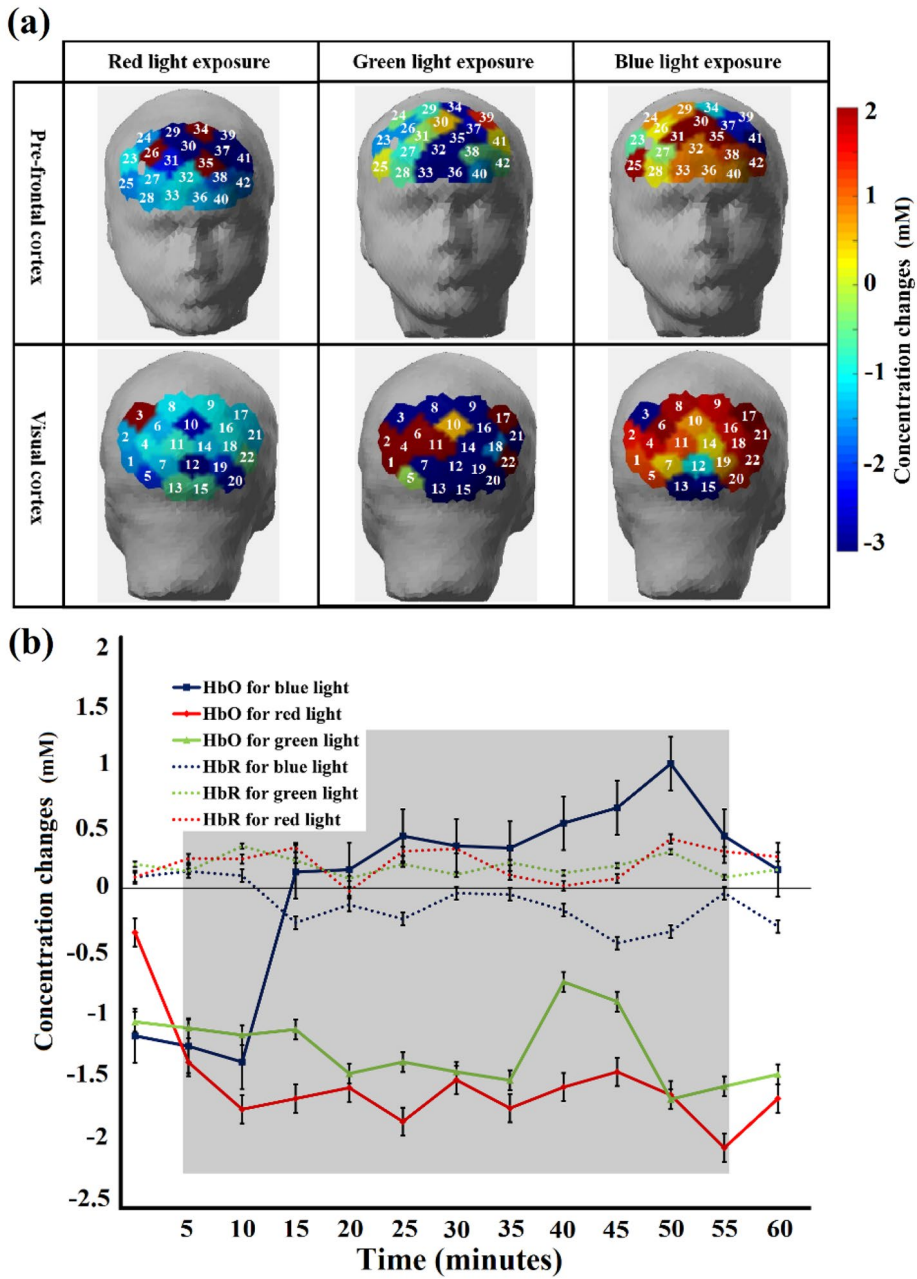
(**a**). The six distinct template maps that best explained channel-wise changes in group mean HbO potential topographies across sleep-deprived conditions for different colors of light exposures (red, green and blue) in prefrontal (upper three in figure) and visual (lower three) cortices. Blue represents a decrease in HbO response, while red illustrates an increase in HbO response. (**b**) Average (group-level of all participants) concentration changes in cerebral oxygenation (HbO and HbR) (mean ± SD) for different colors of light (red, green and blue) during a 60-min drive in a simulator under the sleep-deprived condition. The shaded area represents the time interval during the task/stimulation period (exposure of different colors of light). The time series is subdivided into 5 min each time period. HbO for blue light exposure differed significantly after approximately 15 min of light exposure in comparison with exposure to other colors of light.

By analyzing the neurological signals, it can be deduced that sleep deprivation had a substantial impact on drivers' subjective judgments regarding keeping their safe distance and assessing alertness level. Figure 5a presents the topographic maps of the prefrontal cortex (upper three in figure) and visual cortex (lower three in figure) showing the concentration changes in HbO for different colors of light (red (left two in figure), green (middle two) and blue (right two)). Figure 5b presents the mean concentration changes (mean ± SD) of HbO and HbR for different colors (red, green and blue) of light during a 60-min drive in a simulator under sleep-deprived conditions. Blue light exposure, for the sleep-deprived condition, exhibited an overall statistically significant impact (*p* < α_critical_) on HbO concentrations. An increasing propensity was found for HbO concentrations in response to blue light exposure. Red and green light exposure revealed no notable difference (*p* > α_critical_) in HbO concentration measures compared to the baseline. Conclusively, exposure to blue light contributed to increased total cerebral hemodynamics, increased blood flow, and resultantly higher oxygenation levels in the sleep-deprived condition. Our findings demonstrated greater HbO changes with exposure to blue light compared with the red and green colors of light that conforms to the typical brain activation patterns (topographic maps).

**Figure 6:**
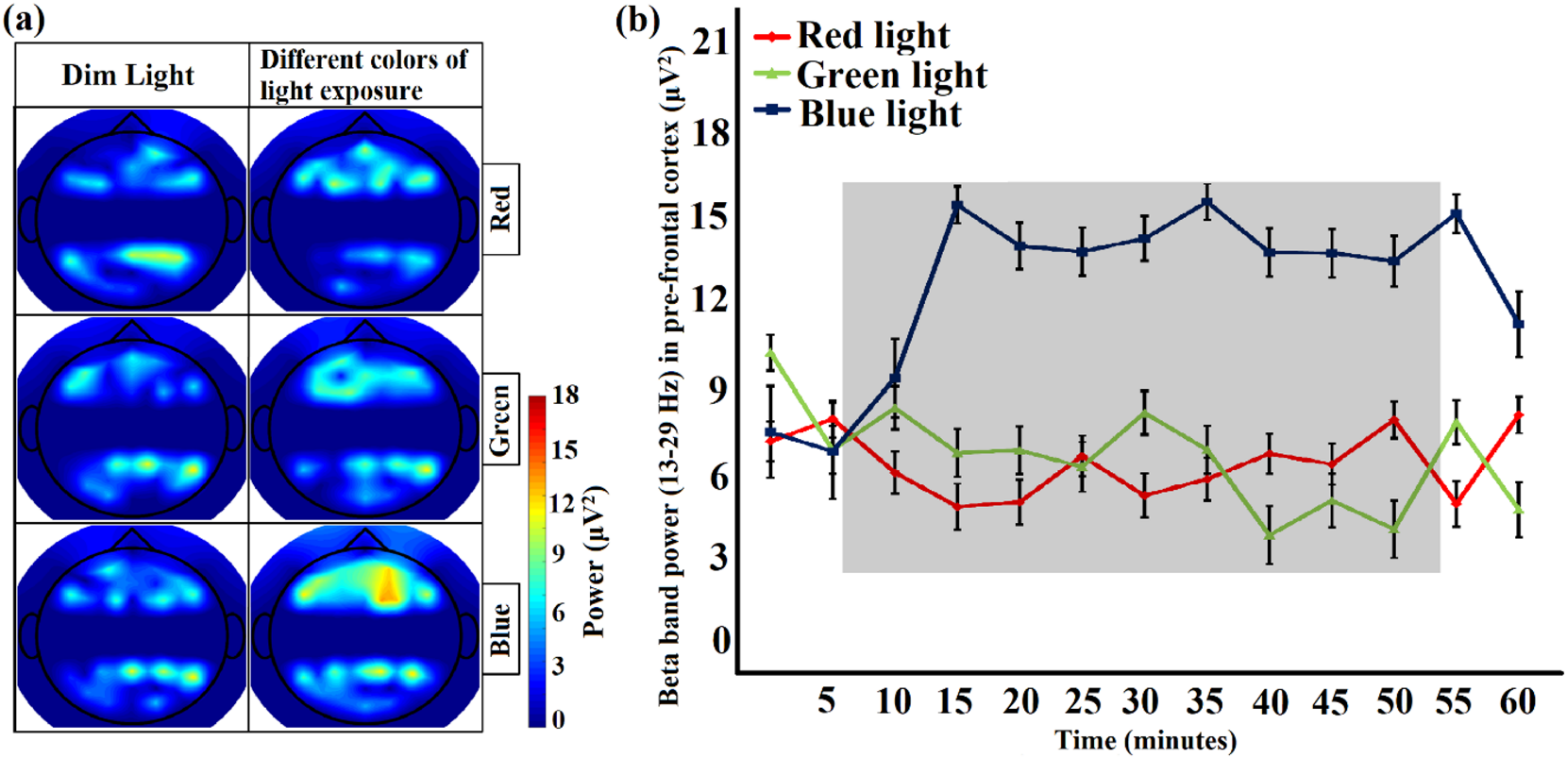
(**a**) The average topography of spatial distributions of EEG beta band power that best explained changes in beta band powers across sleep-deprived and sleep-deprived conditions with exposure to different colors of light. (**b**) Average normalized time course values of the EEG beta band (mean ± SD) for exposure to different colors of light (red, green and blue) under sleep-deprived conditions for pre-frontal cortex region. The shaded area represents the time interval during the task/stimulation period (exposure of different colors of light). The time series is subdivided into 5 min each time period. Drastic discrepancies appear in the EEG beta band after exposure to blue light in contrast with red and green light exposure.

Topographic maps for the beta band power responses against exposure to different colors of light are illustrated in Fig. 6a. In this study, beta band power is the decisive parameter to distinguish awake from sleep deprived states, therefore, mean beta band powers (mean ± SD) for pre-frontal cortex in the sleep-deprived condition are represented in Fig. 6b for each experiment of exposure to colored light. We note that in comparison with exposure to green and red light, only exposure to blue light demonstrated different responses. This figure shows clearly that beta band power increased in cerebral activation with exposure to blue light. In contrast, the other two colors of light (red and green) demonstrated a pattern akin to that of the sleep deprived state.

**Figure 7:**
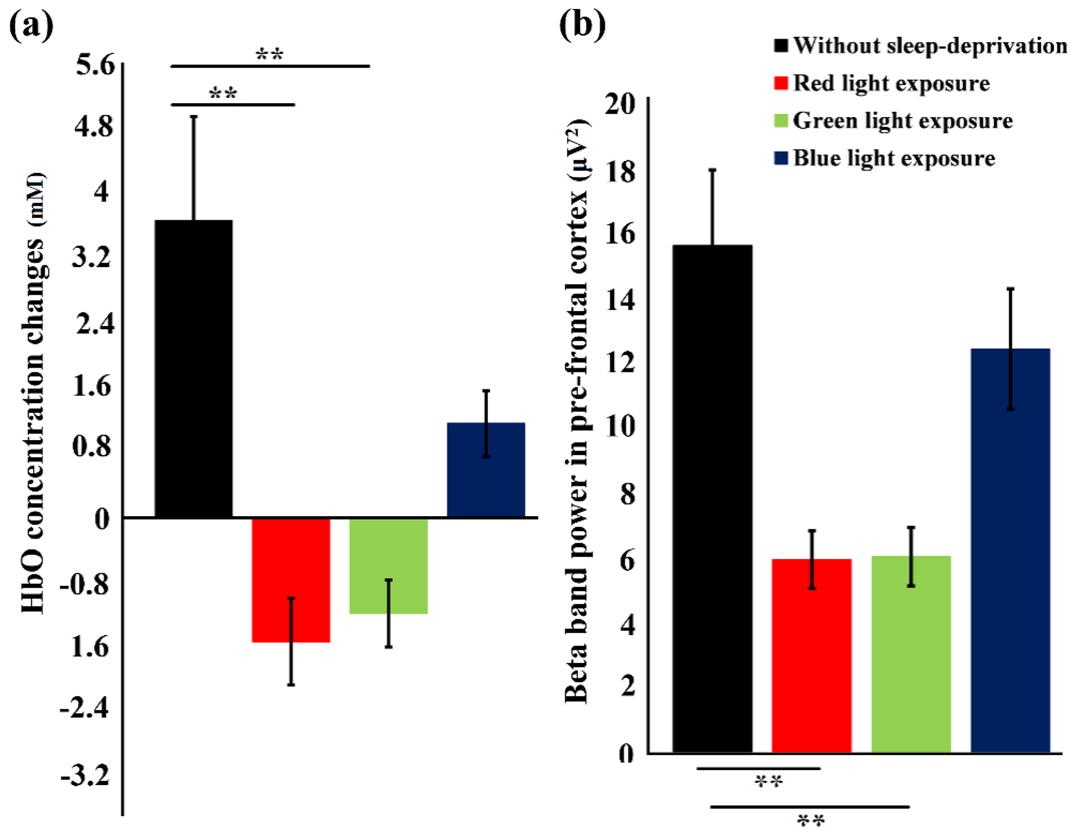
Statistical comparison of (**a**) HbO concentration changes (**b**) and EEG beta band power in pre-frontal cortex. The standard deviation is represented by the error bars. Significant differences in HbO for red and green light exposure and without sleep deprivation data (p < α_critical_) is observed. Similarly, beta band power for red and green light exposure and without sleep deprivation data also show significant differences (p < α_critical_). Whereas, in both HbO and beta band power for blue light exposure shows a trend similar to without sleep deprivation data. ** represented significant difference (p < α_critical_).

In Fig. 7, the mean concentration changes in HbO (Fig. 7a) and the mean beta band power for pre-frontal cortex (Fig. 7b) are displayed for colored light exposure in comparison to the without sleep-deprivation observations. The HbO concentration for blue light exposure increased considerably tending towards the without sleep deprivation response leaving their mutual difference non-significant. However, for red and green light exposures the HbO concentration changes had a significant difference to the without sleep response *p* < α_critical_. Similarly, in EEG analysis, the beta band power under blue light exposure increased, although it was not significant but still, depicting a tendency towards without sleep-deprivation condition. However, the red and green light exposures resulted in a far lesser increase in beta band power and was not significant (*p* = 0.356 and *p* = 0.294 respectively).

**Figure 8:**
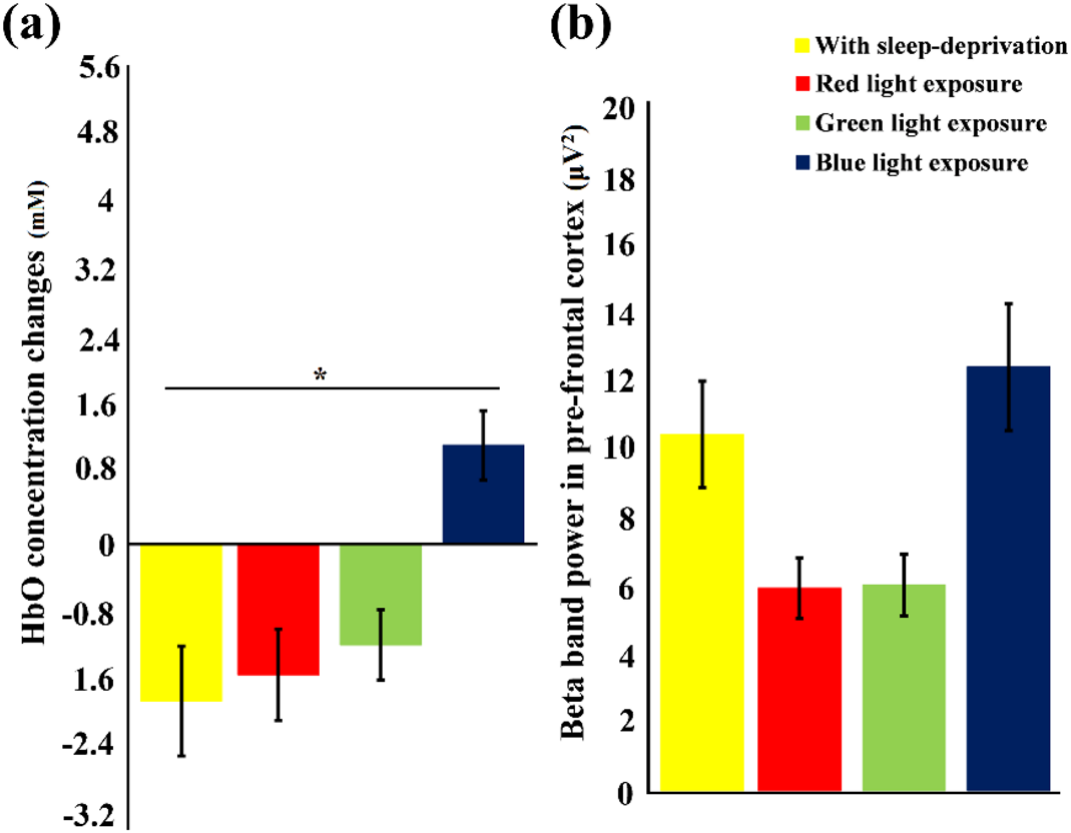
Statistical comparison of (**a**) HbO concentration changes (**b**) and EEG beta band power in pre-frontal cortex. The standard deviation is represented by the error bars. Significant differences in HbO for blue light exposure and with sleep deprivation data (p < α_critical_) is observed. Similarly, beta band power for blue light exposure shows a trend different from sleep deprivation data, although, not significant, but similar to without sleep deprivation data. The visible differences but not significant (p > α_critical_) in all other parameters of fNIRS and EEG for red and green light exposures can be viewed. * represented the significant difference (p < α_critical_).

In Fig. 8, the mean concentration changes in HbO (Fig. 8a) and the mean beta band power for pre-frontal cortex (Fig. 8b) for colored light exposure are displayed in comparison to the with sleep-deprivation observations. Under blue light exposure, the HbO concentration changes increased significantly *p* < α_critical_ in comparison to the with sleep deprivation experiment. Although the increase in HbO under red and green light exposures were also observed but they were not statistically significant (*p* = 0.508 and *p* = 0.471 respectively). Similarly, in EEG analysis, the beta band power under blue light exposure increased although it is not statistically significant (*p* = 0.107) but this increased beta band power depicts a tendency away from the with sleep-deprivation condition and towards the without sleep deprivation condition. Also, under red and green light exposures the increase in beta band power was not significant compared to with sleep deprivation condition (*p* = 0.356 and *p* = 0.294 respectively).

The RM-ANOVA test had a degree of freedom of 90 for corresponding values of participants and the test conditions. The F-score result of RM-ANOVA for test with parameters HbO without sleep deprivation, and HbO after color light exposures was computed to 12.49. Whereas, F-score result of RM-ANOVA for test with parameters EEG beta band of without sleep and EEG beta band of colored light exposures was computed to 15.81. These F-scores provided evidence that the null hypothesis stating that all means are same is not valid and there are at least 2 data groups with statistically significant differences in their means. Further post hoc analyses using Bonferroni confidence interval adjustments were performed. There were a total of 6 comparisons, (3 comparisons of each light (red, green, and blue) exposure responses with the awake brain (results of Experiment-1) state and 3 comparisons of each light exposure responses with the drowsy brain state (results of Experiment-2)), so we divided our critical p-value (α = 0.05) by 6 resulting in an adjusted p-value of 0.0083. The statistical significance thus is decided when the comparison results in a *p*-value < 0.0083 (α_critical_).

**Figure 9:**
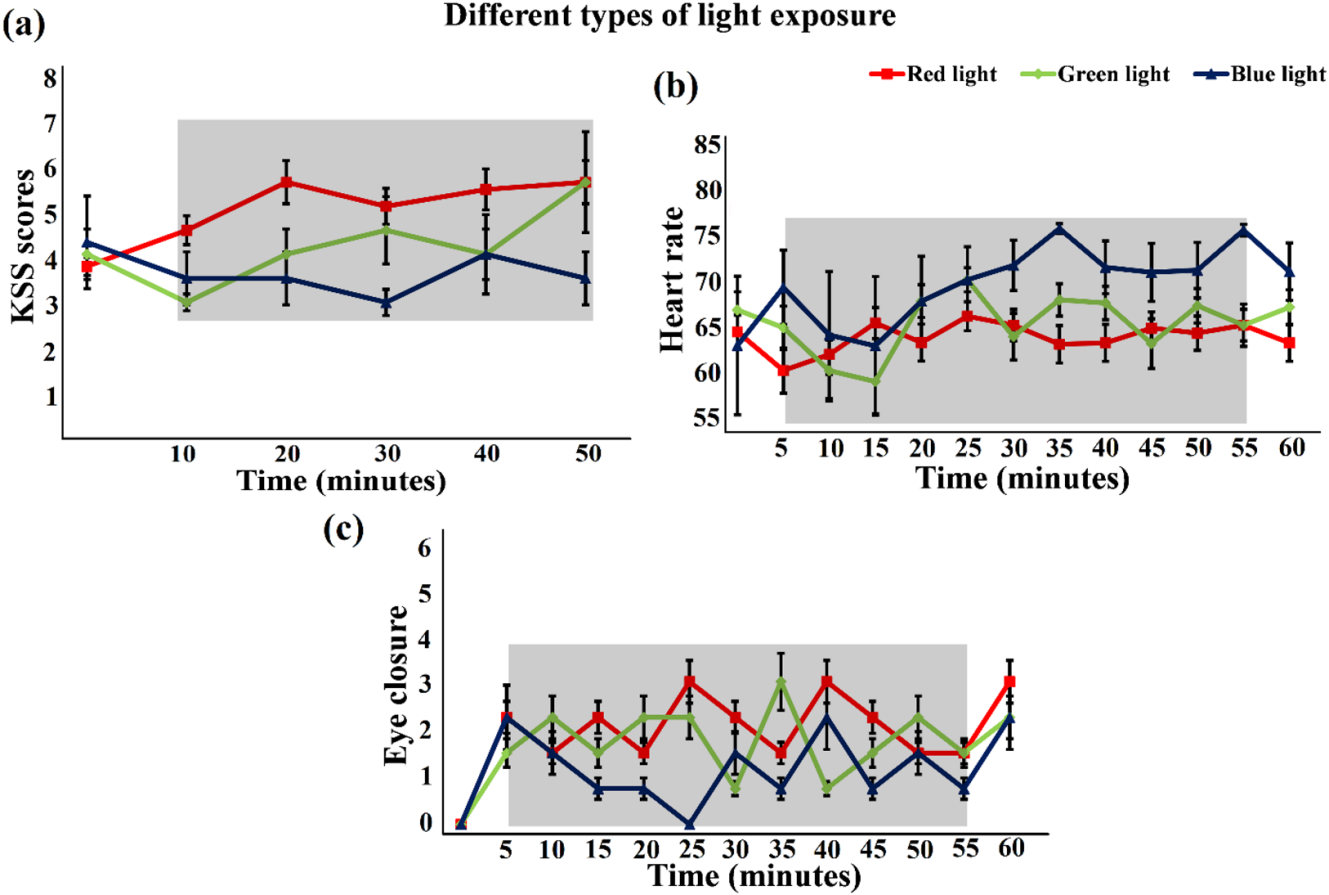
Subjects’ state identification using KSS scores, HR, and EC. The shaded area represents the time interval during the task/stimulation period (exposure to different colors of light). (**a**) KSS scores (mean ± SD) for different colors of light (red, green and blue) under sleep-deprived conditions in 5 time periods (10 min of each time period measured only in stimulation duration) (x-axis) during a 60-min drive in a simulator. (**b**) Average HRs (mean ± SD) of 5-min bins (x-axis) across all participants for different colors of light (red, green and blue) under sleep-deprived conditions during a 60-min drive in a simulator. (**c**) Average EC rates (mean ± SD) of 5-min bins (x-axis) across all participants for different colors of light (red, green and blue) under sleep-deprived conditions during a 60-min drive in a simulator.

Across all participants, the levels of subjective sleepiness on the KSS scale increased significantly in the sleep-deprived state. Figure 4a shows that the average KSS score for well-rested subjects was between 1 and 2 out of 7, but rose to 5 and 6 (sleepy, some effort to keep alert) for sleep deprivation among participants. Notably, there was a progressive increase in the frequency of all subjective symptoms of sleepiness after participants had been awake for approximately 22 h. Figure 9a depicts the average self-reported KSS scores for each period in the colored light exposure experiments. On average, across an hourly experiment of driving under blue light exposure, the KSS score (between 3 and 4) of the participants was found to be close to that of the awake state. At the start of the experiment, the average KSS score of the participants was 3.51, representing a brain state close to neither sleepy nor alert and, it was expected that after a laboriously long driving task, they would be relatively fatigued, with an average KSS score of 6. The average KSS scores in cases of red and green light exposure were reported to be up to 6 showing that red and green light exposures have no considerable effect in retaining the alertness level as compared to the blue light exposure. Alternatively, there may be a chance of false-positives, i.e., the KSS may indicate that a participant is sleep deprived but may be found awake by other methods. Therefore, participants classified as fatigued by the KSS method were validated by matching their physiological data, i.e., EC rate using their recorded video data and HR for accuracy. The mean HRs of the subjects were also calculated according to the exposure to different colors of light over the entire 60-min driving period under sleep-deprived conditions, as depicted in Fig. 9b. As shown in the figure, HRs exposed to blue light were significantly higher than HRs exposed to red and green light. From the video data recorded in the colored light exposure experiments, we found that the average EC rate was lower after exposure to blue light (see Fig. 9c).

### Correlation results

The correlation score was calculated between HbO parameter from the fNIRS signal and beta band of EEG signal with the KSS scores from each of the colored light experiment results. Table 3 presents the correlation scores for each of the corresponding pairs using the MATLAB function corr. The results show that there is a weak positive correlation between HbO and beta band values from the red light exposure with the corresponding KSS values while a weak negative correlation is observed between HbO and beta band values from green light exposure with its corresponding KSS scores. However, a strong negative correlation is found between HbO and beta band values from the blue light exposure with its corresponding KSS values. This shows that as a result of blue light exposure, there is an increase in the HbO and beta band values and a significant decrease in the KSS scores. This decrease in KSS represents a better conscious state of the participant as compared to the that at the start of the experiment.

**Table 3:**
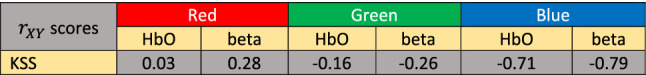
Correlation scores for each color light exposure experiment calculated aginst KSS score of the experiment vs its corresponding HbO and beta values.

## Discussion

The research objective of this study was to evaluate the effects of exposure to red, green, and blue colors of light on human alertness level by assessing blood oxygenation changes (measured with fNIRS), EEG frequency band powers (delta, theta, alpha, beta and gamma) and other physiological measures, including EC rate and HR. This study supports our earlier findings^14^ that, compared with longer wavelength light (red and green) exposure, shorter wavelength light (blue) exposure increases cognitive activity in specific brain regions under fatigued conditions. In the absence of sleep deprivation, the average of all subjects resulted in increased HbO values in comparison to the HbR values, and in the case of sleep deprivation, lower HbO values were observed in comparison to the HbR values. Throughout the 50-min driving experiments, the changes in HbO concentration increased over time in the non-sleep-deprived condition, thus demonstrating high levels in all channels. For the sake of this calculation, every significant pixel was assigned an equal weight. Averaged HbO values were chosen for illustrative purposes since most channels showed similar behavior over time in the prefrontal (upper two in figure) and visual (lower two) cortices for both conditions (sleep-deprived (left two in figure) and non-sleep-deprived (right two). A scientific hypothesis for this study was that exposure to different colors of light will influence subjects' mental state (may convert a sleepy person to an awake state). Fusion of fNIRS and EEG techniques are employed to quantify an individual's state of consciousness (awake or fatigued), and an increase in beta band power (see Fig. 6) and HbO levels (see Fig. 5) induced by blue light exposure represents an increase in alertness level.

EEG provides a number of advantages, such as being noninvasive, compact, low cost, and safe during driving, so most studies to date have utilized EEG for investigating driver alertness, which is a strong predictor of this condition^57^. In one study, researchers observed significant rises in delta, theta, and alpha activity in the fatigue transition process^58^, which was aligned with existing findings according to a review publication^59^. However, the most notable indicator of sleepiness is alpha band activity. Simon et al.^60^ performed a short-term Fourier transformation-based analysis for alpha spindle activity under real-world traffic conditions. Analysis of these actual 20-min driving data has shown that alpha spindle parameters, such as rate, duration, amplitude, and power, have an increasing trend during the transition from the awake state to the drowsy state. In this study, we deduced that the lack of alertness caused by sleep deprivation results in no change in alpha rhythm during simulated driving (see Fig. 3). In the EEG measurements, there was a significant increase in beta band power upon exposure to blue light (see Fig. 6). The normalized beta band power values were introduced to measure fatigue, and a substantial beta band power variation was identified in the targeted brain regions. In earlier studies, it has also been described that an increase in beta band power is closely associated with increased alertness^61,62^, which is potentially useful for safe driving conditions^63^.

In this study, we also used the fNIRS system. The major benefit of the fNIRS system is that it measures oxygen concentration changes in brain blood flow, which is comparable to that in fMRI; therefore, fNIRS is also referred to as portable fMRI with relatively a higher temporal resolution^64^. Additionally, fNIRS may be an important indicator of fatigue detection, and we are certain that using multimodal (fNIRS and EEG) data is advantageous in monitoring alertness. Considering the benefits and limitations of fNIRS technology, blood oxygenation levels throughout the dataset were estimated. From these datasets, a standard criterion was extracted that was used to identify sleep deprived and awake individuals accurately (see section "Identification of the subjects’ state"). Additionally, in the literature, it is also mentioned that the alertness measurement criterion can be represented by oxygen level changes in the prefrontal and visual cortices^65,66^. Since we are using visual stimulation and since the prefrontal cortex is closely associated with mental workload and the enactment of cognitive tasks, we introduced the prefrontal and visual cortices in this work. However, whole head measurements would be more helpful and will be considered for future research.

Several studies have investigated the attributes of light exposure in the brain. Brainard and his colleagues^67^ performed nearly 700 experiments on 72 healthy men and women to identify the most suitable wavelength to inhibit melatonin secretion. This took place from 1995 to 2001. The results corroborated that blue light possesses the most critical wavelengths to regulate the circadian system, which was also demonstrated in a Japanese study on mice^68^. Green color with a wavelength of 555 nm affects the cones called “color receptors”^69^. For the rods, this sensitivity is observed at 507-nm wavelength light^69^. The peak sensitivity of melanopsin receptors spans 459–485 nm, and this argument is supported by previously published research studies on humans, rats, and apes. Light has proven effects on hormone release, HR, body temperature, and gene expression in humans^70^. Blue wavelengths have been found to yield stronger effects than green wavelengths do. Light exposure has effects on EEG beta, alpha, theta, and low-frequency activity, all of which are associated with fatigue level according to earlier research findings^70^. Vandewalle et al.^71^ demonstrated that blue light is more effective than other wavelengths in aiding responses in the left frontal and parietal cortices, both of which play important roles in working memory tasks. In the present study, an abundant increase in the beta band frequency by exposure to blue light is associated with fatigued state effects reduction (see Fig. 6). The significant differences between the sleep-deprived and non-sleep-deprived drive in the beta band (13–29 Hz) applied to differentiate the awake and fatigued states of the brain (see Fig. 3). Additionally, an increase in HR is observed during blue light exposure, but no significant changes in HR are observed during red and green light exposure.

A rise in EEG alpha (theta) power activity and subjective evaluations of alertness were observed in earlier investigations involving continuous driving activity^72–74^. During nighttime, such EEG variations have been connected to risky and worse driving behavior^75^. A positive correlation was found between EEG theta and alpha band spectral powers and alertness level in an experimental study performed on subjects in different age groups using a driving simulator^76^. The reaction time test for young (age range 20–25 years) and older volunteers (age range 52–63 years) who were sleep-deprived was performed in another study by Philip et al.^77^. Although both groups exhibited comparable performance impairment, the young group was found to show a 70 percent decrease, while the senior group showed a decrease of only 15 percent. Elderly subjects had longer (worse) reaction times initially prior to sleep deprivation than their younger counterparts, contributing to other age-related differences pointed out by the study. The research findings highlighted the importance of self-perception of performance and peril among young drivers who usually overstate their driving ability, particularly in sleep-deprived conditions. In this study, the data analysis shows a clear indication of fatigue state reduction when a sleep-deprived subject is exposed to blue light during a 60-min driving simulator experiment (see section "Effects of different colors of light on alertness level"). Furthermore, the KSS mean ratings for blue light exposure are almost 4 on the scale compared to 6 for red and green light exposures (see Fig. 9a); therefore, it is unlikely that blue light exposure can cause a degraded alertness level even after an hour-long driving activity. Hence, in the authors’ opinion, blue light exposure can be useful in managing fatigued state and avoiding accidents by effectively reducing sleepiness.

The experiments reported that the subjects found the driving simulator job quite monotonous, which may be due to the non-haptic simple driving simulator. To evaluate and compare boredom and sleepiness progression in real-world scenarios against this study would be extremely interesting, as it is outside the scope of study. Using a driving simulator offers flexible design parameters, such as changing duration and complexity, and includes other performance metrics, thus accurately reflecting the age-related variations in alertness levels and overall psychomotor function. In future studies, it will be beneficial to study age wise optimal recovery time for long driving stress and to evaluate drivers' response for longer driving hours and higher sleep deprivation times to maximize the risks. It is also important to correctly determine the effects of light on the circadian cycle to develop environments and interventions for healthy circadian rhythms. This study introduced a system to evaluate different color light effects on alertness to anticipate real-world scenarios. This technique has paved the way for the creation and development of a future toolkit that will include personalized circadian medicine to prevent road accidents.

## Conclusion

In this study, the effects of different colors of light on human fatigued states in simulated driving conditions and respective changes in brain hemodynamics in visual and prefrontal cortices were explored by the fusion of EEG and fNIRS. Overall, it may be concluded that an alerting effect of blue light exposure was observed for the beta frequency EEG band and HbO for fNIRS. The results indicate a noticeable reduction in fatigue effects following exposure to blue light in contrast to red and green light exposures. From a practical and human performance perspective, future research suggesting the use of colored light exposure mechanisms should be performed to devise more portable and practical solutions that will make roads and workplaces safer and more productive, respectively.

## Data Availability

The datasets used and/or analyzed during the current study available from the corresponding author on reasonable request.
